# Prognostic value of 2-hour lactate level and lactate clearance for 30-day mortality and comparison with trauma scores in multi-trauma patients

**DOI:** 10.12669/pjms.343.14294

**Published:** 2018

**Authors:** Erhan Altunbas, Serhad Omercikoglu, Haldun Akoglu, Arzu Denizbasi

**Affiliations:** 1 Erhan Altunbas, MD. Department of Emergency Medicine, Atasehir Acibadem Surgery Health Care Center, Istanbul, Turkey. Department of Emergency Medicine, Marmara University Faculty of Medicine, Pendik Education and Research Hospital, Istanbul, Turkey; 2 Serhad Omercikoglu, MD. Department of Emergency Medicine, Marmara University Faculty of Medicine, Pendik Education and Research Hospital, Istanbul, Turkey; 3 Haldun Akoglu, MD. Associate Professor, Department of Emergency Medicine, Marmara University Faculty of Medicine, Pendik Education and Research Hospital, Istanbul, Turkey; 4 Prof. Arzu Denizbasi, MD, PhD. Department of Emergency Medicine, Marmara University Faculty of Medicine, Pendik Education and Research Hospital, Istanbul, Turkey

**Keywords:** Lactate, Lactate clearance,Trauma score, long term mortality

## Abstract

**Objective:**

Trauma scores are prone to misreading. Therefore, a readily available, objective way to estimate the mortality of the trauma patients is needed. We aimed to evaluate the prognostic utility of lactate levels, and clearance for 30-days mortality, and compare with the physiological trauma scores.

**Methods:**

All adult trauma patients (two hundred) admitted to ED were enrolled. Initial and 2-hour serum lactate levels were measured and components of GAP, MGAP, RTS, VIEWS and VIEWS-L trauma scores were calculated.

**Results:**

Final study population was 200 patients with a median age of 33 years. Mortality was 7/200 (3.5%) in 30-days. Both initial (2.3 vs. 7.7 mmol/L) and 2h-lactate (1.7 vs. 8.4 mmol/L) levels were significantly lower, and lactate clearance was significantly higher (23.8% vs. -12.0%) in survivors. Also, the change in the lactate level from 0h to 2h (2.3 vs. 1.7mmol/L) was significant in survivors, contrary to non-survivors (7.7 vs. 8.4mmol/L). VIEWS-L, VIEWS, two hour-lactate level and EMTRAS showed high specificity at the 100% sensitivity cut-offs, therefore, were the most valuable prognostic parameters in this study.

**Conclusion:**

Calculation of 2h-lactate clearance and evaluation of a 2h-lactate level may not be needed to predict long-term mortality if the initial lactate level is below 2.8mmol/L.

## INTRODUCTION

Many scores (Glasgow Coma Scale, Age and Systolic Blood Pressure [GAP], Mechanism and GAP [MGAP], Emergency Trauma Score [EMTRAS], Revised Trauma Score [RTS], VitalPAC Early Warning Score [VIEWS], Modified VitalPAC Early Warning Score with Rapid Lactate Level [VIEWS-L]) were designed to predict short or long-term mortality of the hospital admissions or trauma patients at the initial contact.^1^-^7^ However, validation studies showed conflicting results especially in rural and pre-hospital areas. There are many disadvantages of those scores. Therefore, a readily available, reliable, objective and easy way to estimate the mortality risk of the trauma patients is needed.

Lactate is the final product of the anaerobic metabolism and is a biochemical marker of cellular hypoxia in several shock states.^8^ It is shown to be a diagnostic and prognostic marker in sepsis and trauma, and discriminate major trauma from minor one.^9^ Since, lactate levels increase rapidly in tissue hypoxia and cleared from the blood with restored perfusion and treatment, evaluation of a second lactate level and the rate of decrease (called lactate clearance)were proposed for the estimation of mortality.^10^ However, comparison of the prognostic utility of those clearances with physiological trauma scores were limited.^10^,^11^

Therefore, we aimed to evaluate the prognostic utility of initial and 2^nd^ hour lactate levels, and 2-hour clearance lactate for 30-days mortality, and compare them with the physiological trauma scores.

## METHODS

This is a prospective, observational, prognostic utility study performed at Marmara University Faculty of Medicine, Department of Emergency Medicine (ED). After ethical approval, all trauma patients admitted to trauma bay of the ED between December 2013 and September 2014 were enrolled to the study if they were over 18 years and one of the researchers were on duty. Informed consent was taken from the patient if their condition was stable, in trauma patients who were in criticle conditon, informed consent was taken from his primary relatives. Enrolled patients were excluded if;

1. Trauma scores could not be calculated, or initial lactate level was not obtained at the first 30 minutes of the ED admission.
2. The patient admitted to the ED with the same complaint before.
3. The patient withdraws his/her consent.

All trauma patients were evaluated and managed according to Advanced Trauma Life Support (ATLS) guidelines.^12^ Demographics, vital signs, results of the laboratory examinations, and outcomes of the patients were gathered from the Hospital information system (HIS). Outcome status was confirmed with a follow-up call at the 1^st^ month and via electronic National Surveillance System for the Deceased. Patients with missing parameters were excluded only from the final analysis of those variables.

Hypotension was defined as a systolic blood pressure (SBP) < 90 mmHg according to American College of Surgery.^12^ Venous lactate levels were categorized as 0-2.4 mmol/L (normal), 2.5-4.0 mmol/L (minimally elevated), and >4.0 mmol/L (elevated). BE was categorized as < 0 mEq/L (normal), 0 mEq/L - 6 mEq/L (medium), >6 mEq/L (high). Our hospital is one of the major trauma centers in our city. Our city is divided in regions for trauma patients by our Emergency Medical Systems (EMS). Patients in 20 km around our hospital are carried to our ED by ambulance in maximum 15 minutes. Other patients were excluded from our study. Lactate level of the blood drawn at the first 30 mins of the ED admission was accepted as the initial (0h) lactate level. 2-hour (2h) lactate level was defined as the lactate level at the blood drawn after two hours of the initial test. Lactate clearance was calculated as follows: (0h-Lactate – 2h-Lactate)/ 0h-Lactate x 100.hRTS, GAP, MGAP, EMTRAS, VIEWS, VIEWS-L scores were electronically calculated from the data recorded on HIS. Physicians and researchers were blinded to these calculations. Mortality was defined as the death during the first 30-days of ED admission.

We estimated that 193 patients would be required for a correlation coefficient of 0.2 to be significantly different from 0.0 at a α-level of 0.05 and β-level of 0.20. Therefore, we decided to recruit 200 patients for this study.

Continuous variables were reported as means and standard deviations (SD) with confidence intervals (CI). Categorical variables were reported with frequencies and 25-75 percentile interquartile ranges (IQR). MedCalc Statistical Software version 14 (MedCalc Software bvba, Ostend, Belgium; https://www.medcalc.org) was used for all analysis. The accepted Type-1 and Type-2 errors in this study are 5% and 80% respectively.

## RESULTS

We enrolled 236 patients to the study between December 2013 and September 2014, 36 patients were excluded. Of the study population, 165/200 (82.5%) were male, 185/200 (92.5%) were blunt, and 15/200 (7.5%) were penetrating trauma patients. Median age was 33 years. Majority of patients had motor vehicle accident (MVA) (n=105, 52.5%), and falls (n=63, 31.5%) as the mechanism of their trauma. Nine (4.5%) patients had emergency surgery, 27 (13.5%) patients were admitted to ICU, 54 (27.0%) were admitted to wards and 188 (94.0%) were discharged after ED management. Mortality was 1/200 (0.5%) in the ED, and 7/200 (3.5%) in 30-days.

No significant difference was observed between survivor and non-survivors according to demographic characteristics. SBP, DBP, MAP, SpO_2_, and BE were significantly higher, HR, and INR were significantly lower in survivors (Table-I). Also, in survivors, both 0h- (2.3 mmol/L, IQR:1.7-3.2 mmol/L vs 7.7, IQR: 1.4-2.3 mmol/L) and 2h-lactate (1.7 mmol/L IQR: 1.4-2.3 mmol/L vs 8.4, IQR: 5.8-12.3 mmol/L) levels were significantly lower, and lactate clearance was significantly higher. The difference of the median lactate levels between 0h and 2h were also significant (2.3 mmol/L vs 1.7 mmol/L, p<0.0001, Wilcoxon). However, in non-survivors, there was no significant change in the median lactate level was observed (7.7 mmol/L, IQR:5.5-13.1 vs 8.4 mmol/L, IQR:5.8-12.3, p=0.9375, Wilcoxon. Table I). Mean lactate clearance of the survivors and non-survivors were 23.8% and -12.0%, respectively, indicating an increase in the lactate level of non-survivors. This difference was also statistically significant (Table-I).

**Table-I:** Vital signs and laboratory results of all patients, survivors and non-survivors.

Variable	All Patients (n=200) Median (IQR)	Survivors (n=193) Median (IQR)	Non-survivors (n=7) Median (IQR)	P^1^
GCS	15 (15 to 15)	15 (15 to 15)	12.00 (4.75 to 14.75)	<0.0001
SBP (mmHg)	128.0 (113.5 to 140.0)	130.0 (114.0 to 140.0)	90.0 (84.8 to 117.0)	0.0051
DBP (mmHg)	82.0 (71.5 to 92.0)	84.0 (73.0 to 92.0)	66.0 (50.0 to 75.0)	0.0093
MAP (mmHg)	97.7 (86.7 to 106.7)	98.0 (87.6 to 106.8)	73.7 (64.8 to 88.0)	0.0040
RR (/min)	16.0 (14.0 to 20.0)	16.0 (14.0 to 20.0)	20.0 (15.8 to 24.0)	0.1312
HR (/min)	90.0 (81.0 to 104.0)	90.0 (80.0 to 104.0)	113.0 (88.8 to 121.0)	0.0433
SpO2 (%)	99.0 (97.0 to 99.5)	99.0 (97.0 to 100.0)	89.0 (76.3 to 97.0)	0.0017
INR	1.09 (1.03 to 1.17)	1.08 (1.02 to 1.16)	1.33 (1.19 to 1.45)	0.0003
Base Excess (mEq/L)	0.4 (-2.1 to 1.8)	0.4 (-1.5 to 1.9)	-8.2 (-19.0 to -5.2)	<0.0001
Lactate-1 (mmol/L)	2.4 (1.7 to 3.2)	2.3 (1.7 to 3.2)	7.7 (5.5 to 13.1)	0.0001
Lactate-2 (mmol/L)	1.7 (1.4 to 2.4)	1.7 (1.4 to 2.3)	8.4 (5.8 to 12.3)	<0.0001
Lactate Clearance (%)	23.30 (6.25 to 38.01)	23.81 (7.44 to 39.46)	-12.0 (-22.9 to 14.6)	0.0310

1 Mann-Whitney test, IQR: Interquartile ranges for 25-75 percentiles; statistically significant differences were shown in bold.

When all patients were considered, lactate clearance and trauma scores were not correlated (RTS, GAP, MGAP, EMTRAS, VIEWS and VIEWS-L, Spearman’s rho: 0.18, 0.11, -0.06, -0.13, -0.02, respectively). However, when non-survivors were considered, trauma scores and lactate clearance were shown to be highly and significantly correlated (Spearman’s rho: -0.87, -0.70, 0.56, 0.78, 0.90, respectively. p<0.05 for all). Median RTSc, GAP and MGAP scores were significantly lower, and EMTRAS, VIEWS and VIEWS-L scores were significantly higher in non-survivors, as expected (Table-II).

**Table-II:** Trauma scores of survivor and non-survivors.

Variable	All Patients (n=200) Median (IQR)	Survivors (n=193) Median (IQR)	Non-survivors (n=7) Median (IQR)	P^1^
RTSc	7.841 (7.841 to 7.841)	7.841 (7.841 to 7.841)	6.904 (3.994 to 7.841)	<0.0001
GAP	24.0 (21.0 to 24.0)	24.0 (21.0 to 24.0)	18.0 (13.0 to 19.5)	<0.0001
MGAP	28.0 (26.5 to 29.0)	28.0 (27.0 to 29.0)	22.0 (17.8 to 22.8)	<0.0001
EMTRAS	1.00 (0.00 to 2.00)	1.00 (0.00 to 1.25)	5.00 (4.00 to 5.75)	<0.0001
VIEWS	1.00 (1.00 to 3.00)	1.00 (1.00 to 3.00)	12.00 (8.75 to 15.25)	<0.0001
VIEWS-L	1.35 (1.12 to 3.32)	1.34 (0.47 to 3.18)	13.33 (9.46 to 16.60)	<0.0001

1 Wilcoxon rank-sum test; IQR: Interquartile ranges for 25-75 percentiles;

Statistically significant differences were shown in bold.

All trauma scores, BE, lactate levels and lactate clearance were found to have high prognostic utility for 30-day mortality (Table-III). However, paired comparison of the AUCs revealed no significant difference except BE vs. lactate clearance (difference between AUCs: 0.218, p=0.0128) and 2h-lactate vs. lactate clearance (difference between AUCs: 0.242, p=0.0073). AUCs of initial and 2h-lactate levels and lactate clearance for 30-day mortality were 0.930, 0.982 and 0.740 when all patients were considered. We did not observe any mortality in patients with an initial lactate level of 2.8 mmol/L or less. After the exclusion of those patients, AUC of lactate clearance increased to 0.837 but the difference was statistically insignificant. Dot diagram of the lactate levels and lactate clearance according to survival status is shown in Figure-I. Threshold values to rule-out mortality (sensitivity 100%) were calculated and presented in Table-V. VIEWS-L, VIEWS, 2h-lactate level and EMTRAS showed high specificity at the 100% sensitivity cut-offs, therefore, were the most valuable prognostic parameters in this study.

**Fig.1:**
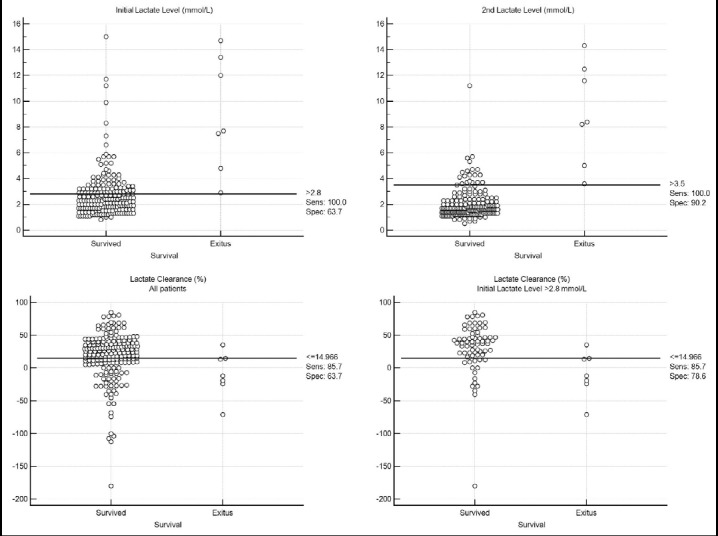
Dot diagrams of lactate levels, and lactate clearance according to 30-day mortality.

All trauma scores, BE, INR, lactate levels and lactate clearance were able to predict need for a stay in the ICU or death. AUCs of RTSc and lactate clearance were statistically significantly lower than VIEWS-L, VIEWS, MGAP, BE and GAP scores (Table-III).

**Table-III:** Comparison of the prognostic accuracies of trauma scores, base excess, lactate levels and clearance for 30-day mortality.

	All Patients		Initial Lactate Level - >2.8 mmol/L (n=77)	

	Accuracy (AUC) (95% CI)	P	Accuracy (AUC) (95% CI)	P
Base Excess	0.958 (0.920 - 0.981)	<0.0001	0.921 (0.837 – 0.970)	<0.0001
Lactate-1	0.930 (0.885 – 0.961)	<0.0001	0.807 (0.701 – 0.888)	0.0183
Lactate-2	0.982 (0.920 – 0.981)	<0.0001	0.962 (0.892 – 0.992)	<0.0001
Lactate Clearance	0.740 (0.674 – 0.799)	0.0061	0.837 (0.735 – 0.911)	<0.0001
RTSc	0.758 (0.693 – 0.816)	0.0160	0.728 (0.614 – 0.823)	0.0479
GAP	0.936 (0.893 – 0.966)	<0.0001	0.908 (0.820 – 0.962)	<0.0001
EMTRAS	0.975 (0.942 – 0.992)	<0.0001	0.951 (0.876 – 0.987)	<0.0001
MGAP	0.928 (0.883 – 0.960)	<0.0001	0.892 (0.800 – 0.951)	<0.0001
VIEWS	0.975 (0.942 – 0.992)	<0.0001	0.952 (0.878 – 0.988)	<0.0001
VIEWS-L	0.981 (0.952 – 0.995)	<0.0001	0.959 (0.888 – 0.991)	<0.0001

* According to the method described by DeLong et al., ** Calculated by exact binomial method.

## DISCUSSION

Our study population was similar to the derivation and validation cohorts of EMTRAS, MGAP and GAP model generating studies according to blunt trauma rate (92.5% vs. 86% to 95%), male ratio (82.5% vs. 68.9% to 72.5%) and mean age (33.0 vs. 38.0 to 51.2). 30-day mortality rate in our study (3.5%) was significantly lower compared to previous studies (15% to 21.9%). In a study by Ahun et al., reported the 30-day mortality rate as 6%, which was closer to our findings.^1^ The lower rate of mortality reported in trauma studies from Turkey is probably associated with the large number of lower acuity patients admitted to trauma centers rather than the ED of community hospitals and family medicine clinics.

We observed that all trauma scores except RTSc were highly accurate for 30-day mortality (Table-III). BE, 0h- and 2h-lactate levels also showed comparable accuracy with those scores (Table-III, Table IV). However, lactate clearance and RTSc score showed lower prognostic accuracy when compared to above. Raum et al. compared RTS, TRISS, NISS and ISS with their newly developed score of EMTRAS in both derivation and validation cohorts for the estimation of in-hospital mortality.^13^ In the derivation cohort, EMTRAS and TRISS was shown to have similar accuracies (0.812, 0.810, respectively), which were significantly higher than RTS, NISS and ISS (0.730, 0.734, and 0.689). In the validation cohort, EMTRAS and TRISS were still more accurate than other scores, however, only TRISS had significantly higher accuracy.^13^

**Table-IV:** Prognostic utility of trauma scores, base excess, lactate levels and clearance for 30-day mortality and threshold values to rule-out mortality in all patients.

	Threshold^1^	Sensitivity	Specificity
Lactate-1 (mmol/L)	≤2.8	100 (56.1-100.0)	63.73 (56.5-70.4)
Lactate-2 (mmol/L)	≤3.5	100 (59-100)	90.16 (85.1-94.0)
Lactate Clearance (%)	≥35.06	100 (59-100)	31.09 (24.6-38.1)
GAP	> 21	100 (59-100)	57.51 (50.2-64.6)
EMTRAS	< 3	100 (59-100)	87.56 (82.1-91.9)
MGAP	> 27	100 (59-100)	53.37 (46.1-60.6)
VIEWS	< 8	100 (59-100)	92.75 (88.1-96.0)
VIEWS-L	<8.53	100 (59-100)	94.30 (90.0-97.1)

1 Calculated from the point with the highest sensitivity on the ROC analysis.

The AUCs of GAP and MGAP scores were reported as 0.87-0.93^1^,^2^, and 0.87-0.94 for the end point of 30-day mortality in several studies.^1^,^13^ We also observed similar findings for GAP (0.936) and MGAP (0.928 and 0.83).

VIEWS is an early warning score derived to be used to predict 24h mortality for inpatients, and AUCs between 0.86 and 0.91 were reported.^14^ We also observed similar AUC at 30-day (0.975) compared to the values reported for 24h. In our study, VIEWS and VIEWS-L scores performed better than other scores as prognostic tools without the need to exclude any patients, which supports the high utility of those scores reported in previous studies.^6^,^7^,^14^

Several studies also investigated the utility of lactate as a prognostic marker. Odom et al. evaluated the predictive value of 6h-lactate clearance in their study, and reported a statistically significant difference between the initial lactate levels of survivor and non-survivors (2.5 and 3.8 mmol/L, respectively).^10^ Odom et al. also reported the AUCs of initial lactate level and lactate clearance as 0.88 and 0.78, and reported both as independent predictors of early (<48h) and late mortality when patients with an initial lactate level of less than 4 mmol/L were excluded.^10^ Regnier et al. also reported the AUCs of initial lactate level and Lactate clearance as 0.78 and 0.70 in all patients.^11^ In our study, AUCs of initial and 2h-lactate levels and lactate clearance for 30-day mortality were 0.930, 0.982 and 0.740 when all patients were considered. We did not observe any mortality in patients with an initial lactate level of 2.8 mmol/L or less (Table-IV). After the exclusion of those patients, AUC of increased to 0.837, which was higher than the reported values by Odom et al. In our study, AUC of initial lactate level was higher compared to lactate clearance, similar to previous studies.^10^,^11^ We think that, the reason for the relatively lower AUC of lactate clearance when all patients were included is a mathematical relativity problem arising from the calculation method. When the initial lactate level is already low, the relative change (lactate clearance) may be high despite the insignificant change in the absolute value. We think that this problem decreases the utility of lactate clearance especially in patients with extremely high and low initial lactate levels. This point was also recognized and discussed previously by Regnier et al.^11^ They stated that “the meaning of lactate clearance may not be the same in patients with normal initial blood lactate level.”^11^

Claridge et al. reported the mortality as 100% if lactate levels fail to decrease.^15^ However, in our study, only 4 of the 47 (8.5%) patients with an increase in lactate level had mortality in 30-days. We think that for the calculation of lactate clearance two hours may not be accurate enough to predict 30-day mortality.

Therefore, calculation of a 2h-lactate clearance and evaluation of a 2h-lactate level may not be needed to predict long term mortality if the initial lactate level is 2.8 mmol/L or less. By reserving lactate clearance for those with a higher initial lactate level, long-term predictive value of this parameter seems to be increased. This approach eliminates the need for the calculation of trauma scores to predict 30-day mortality since initial lactate level and clearance exhibit similar prognostic utilities.

### Limitations

We had to exclude 15% of the enrolled patients according to exclusion criteria from the study, and the most common reasons were the lack of data for the components of the scores and initial lactate level. The unavailability of the data was probably due to the high acuity and severity of those trauma patients. This may have affected our final mortality rate and enrollment of high risk patients into our study population.

## CONCLUSION

Calculation of 2h-lactate clearance and evaluation of a 2h-lactate level may not be needed to predict long term mortality if the initial lactate level is 2.8 mmol/L or less.

### Authors’ Contributions

**EA and HA** conceived the study and designed the trial.

**EA, SO, AD, HA** supervised the conduct of the trial and data collection.

**EA and HA** undertook recruitment of participating patients and managed the data, including quality control.

**HA** provided statistical advice on the study design and analyzed the data.

**AD** chaired the data oversight committee.

**EA, SO, AD, HA** drafted the manuscript, and all authors contributed substantially to its revision.

**EA** takes responsibility for the paper as a whole.
